# Case Report: SAPHO Syndrome Mimicking Bone Metastases During Treatment With Pembrolizumab for Non-small Cell Lung Cancer

**DOI:** 10.3389/fmed.2021.679111

**Published:** 2021-07-22

**Authors:** Yuko Kubo, Kimiteru Ito, Yutaka Fujiwara, Tatsuya Yoshida, Masahiko Kusumoto

**Affiliations:** ^1^Department of Diagnostic Radiology, National Cancer Center Hospital, Tokyo, Japan; ^2^Department of Thoracic Oncology, National Cancer Center Hospital, Tokyo, Japan

**Keywords:** pembrolizumab, SAPHO syndrome, immune-checkpoint inhibitors, immune- related adverse event, non-small cell lung cancer

## Abstract

A 69-year-old female with recurrent stage IV squamous cell lung carcinoma and metastatic abdominal lymph node but not bone metastases was being treated with pembrolizumab. Four months after starting the recurrent treatment, the tumour reduced in size but she began to complain of back pain and palmar rash. A bone scan showed uptake lesions in the left sternocostal joints and vertebrae, while spine magnetic resonance imaging (MRI) showed multiple lesions in the thoracic vertebrae. Her heterogeneous lesions, such as skin and multiple bone manifestations, were comprehensively diagnosed as SAPHO syndrome by different experts. Furthermore, the SAPHO syndrome was suspected to be an immune-related adverse event induced by pembrolizumab, and pembrolizumab withdrawal and prednisolone treatment were performed. Subsequently, her symptoms improved and the follow-up imaging findings showed that the bone lesions had almost disappeared. This case demonstrates that SAPHO syndrome mimicking bone metastases developed during treatment with pembrolizumab. SAPHO syndrome is rare and bone lesions related to the disease may be misdiagnosed as bone metastases. Therefore, it is important in the future for various physicians to have a better understanding of SAPHO syndrome and to consider the potential relationship between this disease and immunotherapy.

## Introduction

Non-small cell lung cancer (NSCLC) encompasses 85% of total lung malignancies, which remains to be the leader malignancy worldwide, accounting more than 1.8 million of deaths in 2020 (1). In the last decade, one of the major advances in the diagnosis and treatment of lung cancer has been the improved knowledge of oncogenic driver mutations and immune-checkpoint inhibitors (ICIs) that have revolutionised therapeutic strategies. Immunotherapy has been shown to induce robust responses in patients with advanced cancer (2). Accordingly, pembrolizumab, a humanised IgG4 monoclonal antibody against programmed cell death protein 1 (PD-1), showed antitumor activity in patients with advanced NSCLC by disrupting the binding of PD-1 to its ligand and impeding the inhibitory signals in T cells (3). Despite important clinical benefits, pembrolizumab has been associated with a wide spectrum of side effects termed as immune-related adverse events (irAEs) that occur as a consequence of general immunological stimulation (4). SAPHO syndrome is a rare disease of unknown aetiology, and few cases of concomitant disease during the course of lung cancer treatment have been reported (5, 6). We herein describe a case of SAPHO syndrome mimicking bone metastases during treatment with pembrolizumab in a patient with stage IV NSCLC. We present the following case in accordance with the CARE Guideline (7).

## Case Description

**Figure 1:**
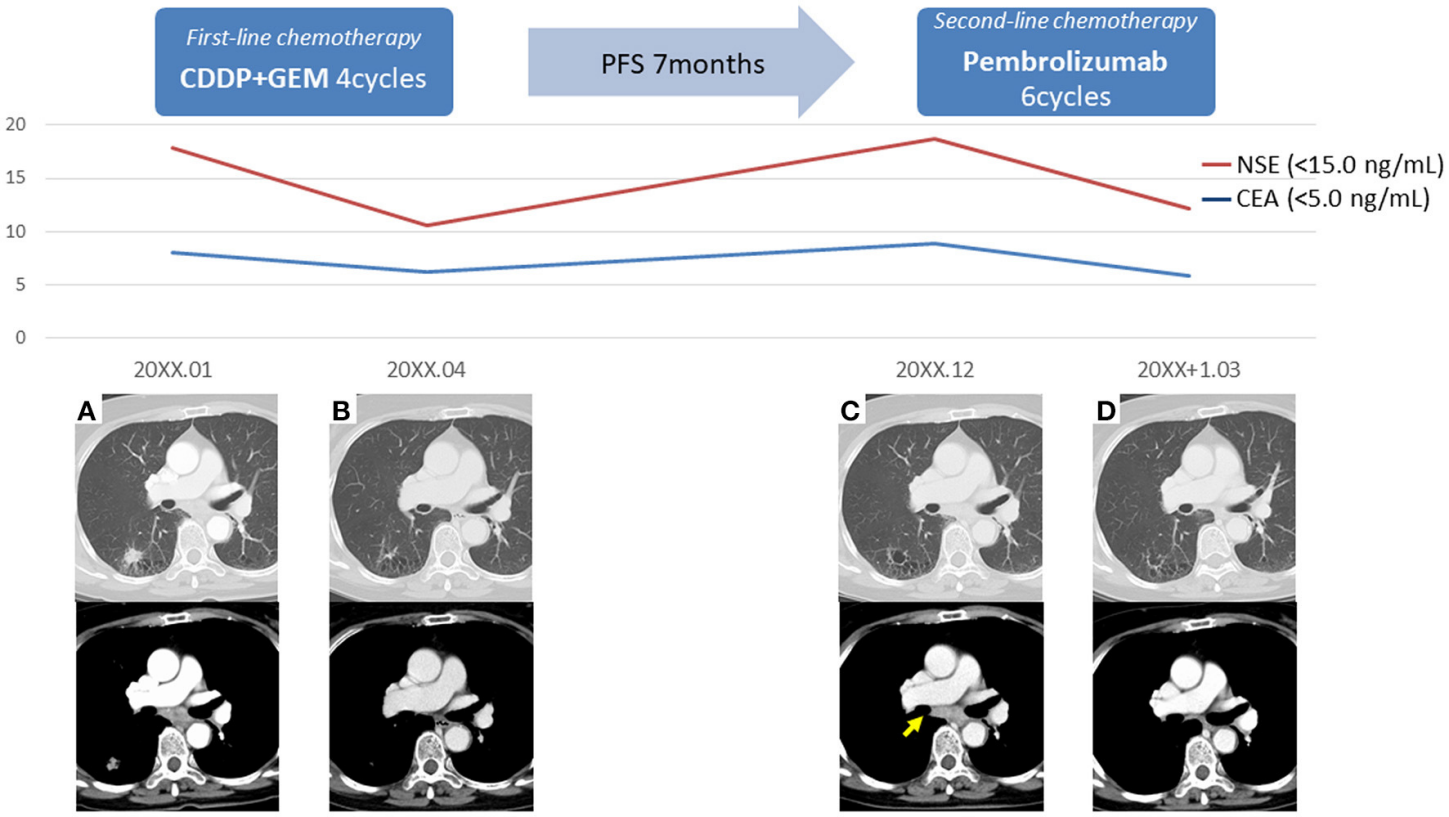
Timeline of computed tomography images during the therapeutic course. **(A)** The initial images at the time of diagnosis, **(B)** The images after first-line chemotherapy showed reduction of right lower lobe lung cancer and mediastinal lymph node metastasis, **(C)** The images after 7 months of first-line chemotherapy showed lymph node recurrence (arrow), **(D)** The images after second-line chemotherapy showed reduction of mediastinal lymph node metastasis; PFS, progression-free survival.

**Figure 2:**
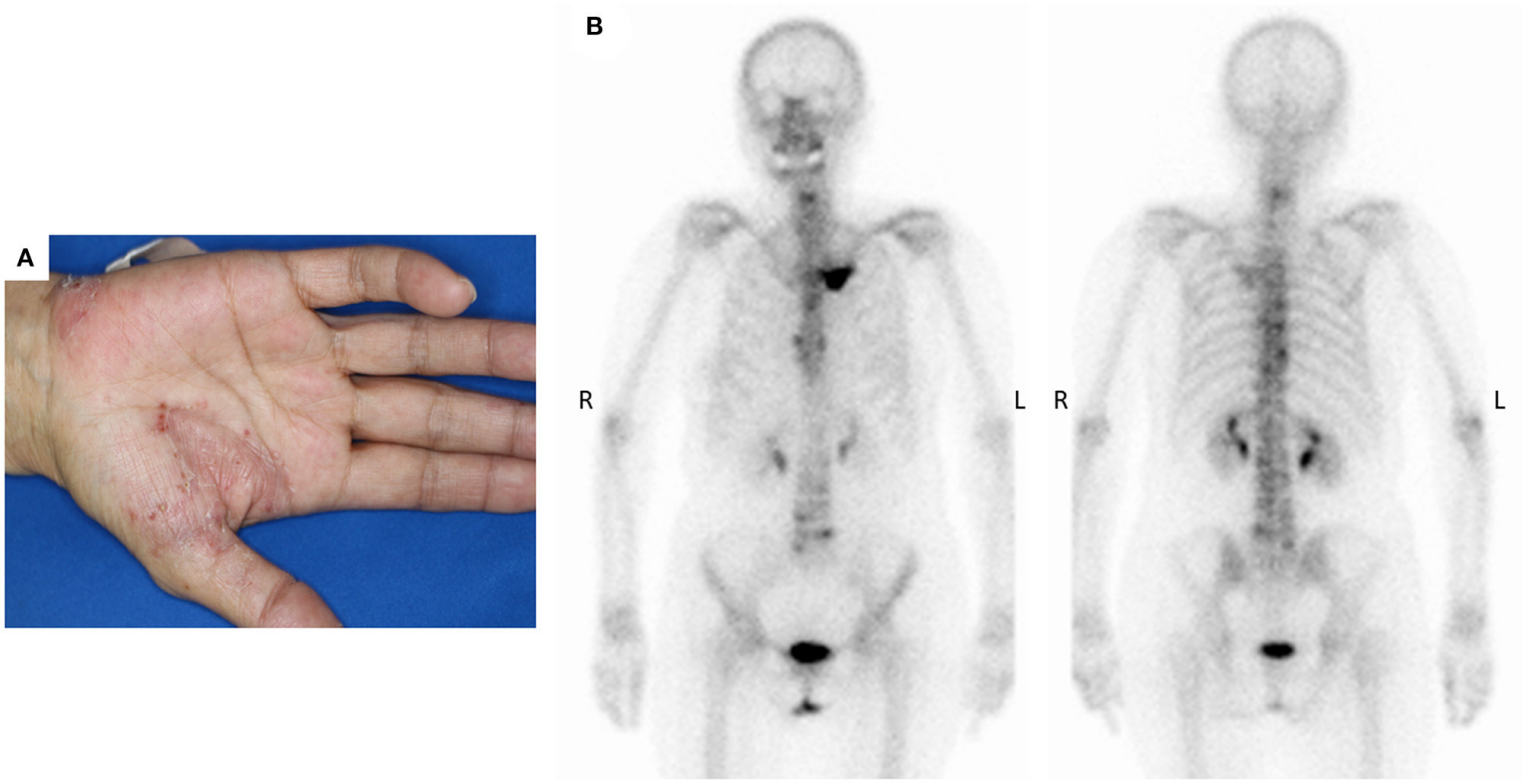
**(A)** The patient developed palmoplantar pustular skin lesions after 4 months of pembrolizumab immunotherapy, **(B)** Bone scan detected multiple abnormal sites of uptake at the left sternoclavicular joints and across multiple vertebrae.

**Figure 3:**
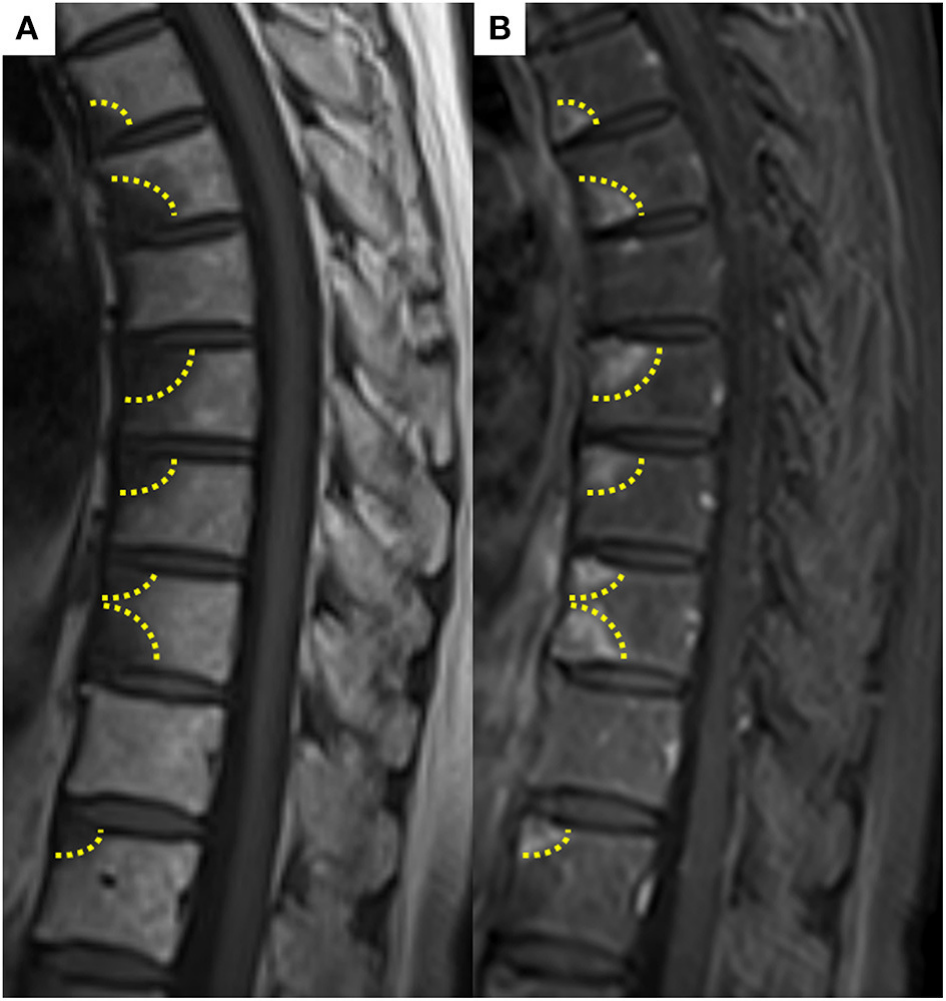
**(A)** Spine magnetic resonance imaging showed hypointense area on T1-weighted image, **(B)** abnormally enhancing effect on contrast T1-weighted image in contiguous anterior thoracic vertebral corners. These multiple vertebral lesions had a characteristic semi-circular pattern (dashed line) in contiguous vertebral body segments.

The patient was a 69-year-old woman with a history of smoking who received cisplatin and gemcitabine as first-line chemotherapy for stage IV squamous cell lung cancer with abdominal lymph node metastasis but no bone metastasis. Her cancer temporarily disappeared due to the effects of first-line chemotherapy; however, the lymph node metastasis recurred 7 months later and she received second-line chemotherapy with pembrolizumab. Timeline of computed tomography (CT) images during the therapeutic course was shown in Figure 1. Four months after starting the treatment, the tumour had reduced in size, but the patient developed severe back pain and a rash on both palms resembling pustulosis palmoplantaris (Figure 2A). A Tc-99m methylenediphosphonate bone scan was performed in suspicion that her back pain was due to bone metastasis. Accordingly, the bone scan revealed abnormal uptake in multiple vertebrae and left sternoclavicular joint (Figure 2B). Subsequently, MRI was performed to closely evaluate the vertebral lesions, showing multiple bone lesions with a hypointense area on T1-weighted images and contrast-enhancing effects on contrast T1-weighted images (Figure 3). These multiple vertebral lesions had a characteristic pattern of SAPHO syndrome showing a semi-circular pattern of contiguous vertebral body involvement localised at the anterior vertebral corners, unlike the usual bone metastases.

**Figure 4:**
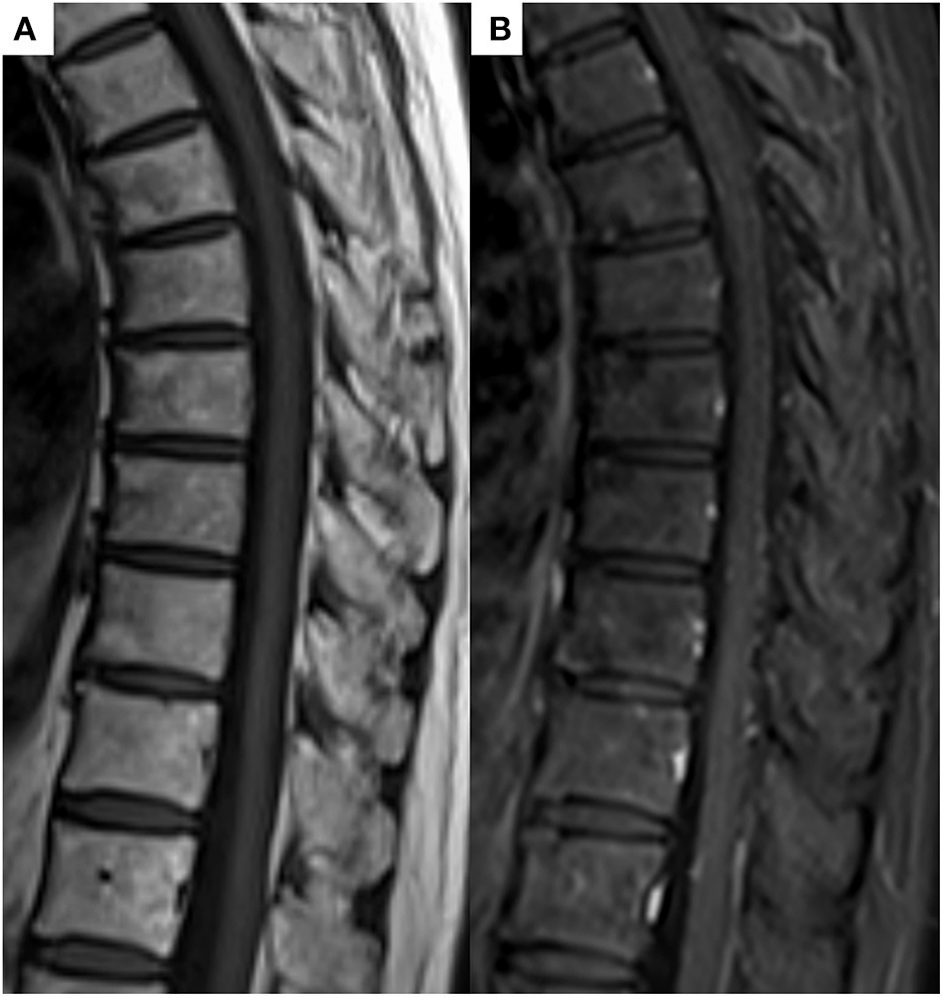
**(A)** Follow-up T1-weighted image and **(B)** contrast T1-weighted image after 5 months showed complete resolution of all previously noted bony lesions.

In conclusion, the patient was comprehensively diagnosed with SAPHO syndrome by different experts, including dermatologists, thoracic oncologists, and radiologists, based on the diagnostic criteria proposed by Benhamou (8, 9) because although the cancer was reduced after pembrolizumab treatment, new heterogeneous lesions such as osteoarticular manifestations with palmoplantar pustulosis were observed. Five months after the discontinuation of pembrolizumab and immunosuppression therapy with prednisolone, the follow-up MRI showed complete resolution of all previously observed multiple bone lesions (Figure 4). Follow-up Tc-99m methylenediphosphonate bone scan after 6 months showed decreased uptakes in the left sternoclavicular joint and multiple vertebras. At present, the patient has remained cancer-free, about 3 years after pembrolizumab withdrawal; however, she continues to be medicated prednisone due to joint pain.

## Discussion

SAPHO syndrome, first identified in 1987 by Chamot et al. is a disorder of unknown aetiology that is manifested by synovitis (inflammation of the joints), acne, pustulosis (thick yellow blisters containing pus) often on the palms and soles, hyperostosis (increase in bone substance) and osteitis (inflammation of the bones) (10). There are several published diagnostic criteria for SAPHO and the presence of only one of the inclusion criteria is sufficient for diagnosis. However, the criteria suggested by Kahn and others by Benhamou (8, 9) have been applied most frequently. The clinical presentation of SAPHO syndrome is heterogeneous, so patients may be examined by different experts to reach a comprehensive diagnosis, although sometimes reaching a diagnosis is difficult and a biopsy may be needed. In this case, heterogeneous clinical presentation, such as osteoarticular manifestations with palmoplantar pustulosis, was comprehensively diagnosed by different experts, including dermatologists, thoracic oncologists and radiologists. Since the syndrome has radiological characteristics, these are useful and recognisable in diagnosis using X-ray, CT, MRI and bone scintigraphy. Uptake in the sternoclavicular region shown in bone scintigraphy is characteristic of the SAPHO syndrome (11). Previous reports have shown that SAPHO syndrome can be difficult to differentiate from multiple bone metastases associated with lung cancer (5). However, other reports have showed that the semi-circular pattern of vertebral body signal alteration and enhancement may help differentiate SAPHO syndrome from metastases, which tend to be randomly distributed throughout the spine (12). A recent review has reported that FDG PET/CT may be useful in differentiating SAPHO syndrome (13). In our case, the characteristic radiological findings on bone scintigraphy and MRI supported the diagnosis of SAPHO syndrome.

Pembrolizumab is a selective humanised IgG4 kappa monoclonal antibody that inhibits the PD-1 receptor, an integral component of immune-checkpoint regulation in the tumour microenvironment. PD-1 is directly involved in the peripheral tolerance of self-reactive T cells. Immune checkpoints are necessary to maintain a balance between activation and quiescence of the immune system and suppress self-reactive T cells. By blocking these checkpoints, ICIs disrupt this delicate balance, which may cause autoimmune events. A recently published multicentre retrospective analysis reported that pembrolizumab-induced irAEs occurred in approximately one-third of all cases, with cutaneous disease being the most common followed by rheumatic irAEs in 12.6% of the cases (14).

The SAPHO syndrome can affect patients of any age, and its aetiology is still unknown. The pathogenesis of SAPHO may be multifactorial, involving a combination of genetic, infectious and immunological components. One aetiology of SAPHO syndrome is thought to involve innate immunologic components (15), with a case of bacillus Calmette-Guérin vaccine immunotherapy-induced SAPHO syndrome having been documented (16). Recent literature has reported a high incidence of malignancy in SAPHO syndrome (17). However, to the best of our knowledge, no previous study has reported on SAPHO syndrome during treatment with pembrolizumab for non-small lung cancer. Furthermore, in this case, withdrawal of pembrolizumab and administration of steroids dramatically and reversibly improved the clinical findings. Thus, we considered that immunotherapy with pembrolizumab could be one of the possible aetiologies of inducing SAPHO syndrome as rheumatic irAEs (12).

This case report demonstrates that SAPHO syndrome mimicking bone metastases developed during treatment with pembrolizumab. SAPHO syndrome is a rare syndrome, with bone lesions related to the disease being misdiagnosed as bone metastases. Therefore, it is important in the future for various physicians to have a better understanding of SAPHO syndrome and to consider the potential relationship between this disease and immunotherapy.

## Data Availability Statement

The original contributions presented in the study are included in the article/Supplementary Material, further inquiries can be directed to the corresponding author/s.

## Ethics Statement

Written informed consent was obtained from the individual(s) for the publication of any potentially identifiable images or data included in this article.

## Author Contributions

YK: conceptualisation, data curation, project administration, visualisation, investigation, writing—original draft preparation, writing—reviewing, and editing. KI: conceptualisation, visualisation, and investigation. YF and TY: data curation and supervision. MK: supervision and writing—reviewing and editing. All authors contributed to the article and approved the submitted version.

## Conflict of Interest

YF received grants and personal fees from Astra Zeneca, grants from Abbvie, grants and personal fees from Bristol-Myers Squibb, grants and personal fees from Chugai Pharma, grants and personal fees from Daiichi Sankyo, grants from Eisai, grants from Eli Lilly, grants from Incyte, grants from Merck Serono, grants and personal fees from MSD, grants and personal fees from Novartis, personal fees from Ono Pharmaceutical, outside the submitted work. TY has received grants from Ono Pharmaceutical, Bristol-Myers Squibb, AstraZeneca, Takeda, AMGEN, Abbvie, Daiichi-Sankyo and MSD and has received personal fees from AstraZeneca, Chugai, Novartis, Archer DX, Boehringer Ingelheim, Lilly, Bristol-Myers Squibb and MSD. MK received honoraria for lecture fees from Ono pharmaceutical Co.,Ltd, AstraZeneca K.K. and MSD K.K. MK received a research grant from Canon medical systems corporation. The remaining authors declare that the research was conducted in the absence of any commercial or financial relationships that could be construed as a potential conflict of interest.
